# Supplementary material for: Patterns of Intron Gain and Loss in Fungi
Source: PLoS Biol. 2004 Nov 30;2(12):e422. doi: 10.1371/journal.pbio.0020422 (PMC532390; doi:10.1371/journal.pbio.0020422)
Supplement: Table S1 — Also available at http://genes.mit.edu/NielsenEtAl/. (4.3 MB ZIP). [file pbio.0020422.st001.zip › NielsenEtAl/html/83.html]

AN5121.1.NCU01547.1.MG01059.1.FG10738.1


```
 CLUSTAL W (1.82) Multiple Sequence Alignments - Introns Inserted


Sequence 1: NCU01547.1	352 aa
Sequence 2: MG01059.1	357 aa
Sequence 3: FG10738.1	344 aa
Sequence 4: AN5121.1	344 aa
Alignment Length: 365 aa
Number Identitical Residues: 234 aa
Alignment Score (without introns) 9535


MG01059.1 	MPSTTAETLSLVTRNVSVAPLVLLSVVDHYNRESANQTKNKRVVGVLLGQNDGKNVRVSN
NCU01547.1	MPATTAETLSLVNRSVSVAPLVLLSVVDHYNRTQANKSKSKRVVGVLLGQNDGKNVRVSN
FG10738.1 	MPTTTAETLSLVTRNVTVAPLVLLSAVDHYNRTVSTKTK-RRVVGVLLGQNDGNDVRVSN
AN5121.1  	MPATTADTLSLVTRTVTVAPLVLLSVADHYGR--SAKGTRKRVVGVLLGEHIGGNVRVSN
          	**:***:*****.*.*:********..***.*  : : . :********:: * :*****

MG01059.1 	SFAV1PFEEDEKNPSVWFLDHNYVESMNDMFKKVNAREKLIGWYHSGPKLRASDLEINEL
NCU01547.1	SFAV1PFEEDDKDPSVWFLDHNYVESMNDMFKKVNAREKLIGWYHSGPKLRASDLDINEL
FG10738.1 	SFAV1PFEEDDKDPSVWFLDHNYVESMNDMFKKVNAREKLIGWYHTGPKLRASDLEINEL
AN5121.1  	SFAV1PFEEDEKDPSVWFLDHNFVESMRDMFKKINAREKLIGWYHSGPKLRASDLEINEL
          	**** *****:*:*********:****.*****:***********:*********:****

MG01059.1 	FKRYTPNPLLVIIDVQPKEAGVPTDAYFAVDEIKD0DGTATSRTFVHTPSIIEAE~EAEE
NCU01547.1	FKRYTPNPLLVIVDVQPKETGVPTDAYFAVDEIKD0DGTTTSKTFVHTPSIIEAE~EAEE
FG10738.1 	FKRYTPNPLLVIVDVQPKESGVPTDAYFAVDEIKD0DGTTTARTFVHTPSVIEAE~EAEE
AN5121.1  	FKRYTPNPLLVIVDVQPKEVGVPTDAYFAVDEIKD~VGTTAPQ-----PRHTLTL0EAEE
          	************:****** ***************  **::.:     *    :  ****

MG01059.1 	IGVEHLLRDIRDVAVGTLSTRLTNQLQSLQGLHLRLRDIQAYLQKVLDGDLPVNHAILGN
NCU01547.1	IGVEHLLRDIRDVAVGTLSTRITNQMRSLQGLHHRLRDIQAYLQKVLDGQLPVNHAILGN
FG10738.1 	IGVEHLLRDIRDVAAGTLSTRVTNQLQSLQGLHLRLRDIGAYLQKVLDKQLPINHAILGN
AN5121.1  	IGVEHLLRDIRDVAVGTLSTRITSQLQSLQGLHLRLRDIGQYLQKVLDHELPVNHAILGN
          	**************.******:*.*::****** *****  ******* :**:*******

MG01059.1 	LQDVFNLLPNLSTPSGGKGSG--------DGGLEHAMAVKTNDQLMSIYLSSLIRAITAF
NCU01547.1	LQDVFNLLPNLSTPKSGPGATG----TNADSELNHAMSIKTNDQLMAIYLSSLIRAITAF
FG10738.1 	LQDVFNLLPNLSTPERDGKSG--------GGELAYAMSVKTNDQLMAIYLSSLIRAITAF
AN5121.1  	LQDVFNLLPNLSTPSTTARVGGEPEQSTDNAELARAMSIKTNDQLMAIYLSSLVRAVTAY
          	**************.      .....:. .. *  **::*******:******:**:**:

MG01059.1 	HDLIENKIQNRQQQEEKEAAKKEGEKDAKDGDKKEDGKGAVNGVNGDGPAKEGASDKDKE
NCU01547.1	HDLIENKIQNRQQQEENDAKKKEGE----NGEKKE----GADKKEGSPAAANGES-KEKE
FG10738.1 	HDLIENKIQNRQQQEEKEAKKEEINN---KDEKKE----GTNG--ASTQAKEGGE-KDKE
AN5121.1  	HDLIENKIQNRQQQEEVEQKREQEAN----SAKNE----------KEGAKKVTNTSEPKE
          	**************** :  :::  .    . *:*           .        .: **

MG01059.1 	AGSKDKKK
NCU01547.1	NSPKEKKK
FG10738.1 	N--KETKK
AN5121.1  	ESKEKTKR
          	 . :..*:
```
